# Zone-Based Routing Protocol for Wireless Sensor Networks

**DOI:** 10.1155/2014/798934

**Published:** 2014-11-18

**Authors:** Muni Venkateswarlu Kumaramangalam, Kandasamy Adiyapatham, Chandrasekaran Kandasamy

**Affiliations:** ^1^Department of Mathematical and Computational Sciences, National Institute of Technology Karnataka, Mangalore 575025, India; ^2^Department of Computer Science and Engineering, National Institute of Technology Karnataka, Mangalore 575025, India

## Abstract

Extensive research happening across the globe witnessed the importance of Wireless Sensor Network in the present day application world. In the recent past, various routing algorithms have been proposed to elevate WSN network lifetime. Clustering mechanism is highly successful in conserving energy resources for network activities and has become promising field for researches. However, the problem of unbalanced energy consumption is still open because the cluster head activities are tightly coupled with role and location of a particular node in the network. Several unequal clustering algorithms are proposed to solve this wireless sensor network multihop hot spot problem. Current unequal clustering mechanisms consider only intra- and intercluster communication cost. Proper organization of wireless sensor network into clusters enables efficient utilization of limited resources and enhances lifetime of deployed sensor nodes. This paper considers a novel network organization scheme, energy-efficient edge-based network partitioning scheme, to organize sensor nodes into clusters of equal size. Also, it proposes a cluster-based routing algorithm, called zone-based routing protocol (ZBRP), for elevating sensor network lifetime. Experimental results show that ZBRP out-performs interims of network lifetime and energy conservation with its uniform energy consumption among the cluster heads.

## 1. Introduction

Wireless sensor network (WSN) is a distributed collection of resource constrained tiny nodes capable of operating with minimal user attendance. Rapid development in microelectromechanical systems (MEMS) technology has provided small sized, low-power, and low-cost sensor nodes with the capability to sense various types of physical and environmental conditions. WSN improves the ability of humans to monitor and control physical locations from faraway places [1]. Since each sensor node works independently without any central control, failure of some nodes does not affect other network activities. WSN is more reliable and secure when compared to other existing types of networks. WSN is the backbone for establishing smarter environment. Each sensor node is equipped with one or more low powered sensors, a processor, memory, a power supply, a radio, and an actuator [2]. Based on infrastructure, WSNs are of two types: structured WSNs and unstructured WSNs. Nodes are deployed in predetermined way in structured WSN, whereas in unstructured WSN they are randomly deployed. Usually, structured WSN has densely deployed sensor nodes, which are not easily manageable and unstructured WSN will have limited number of sensor nodes which can be easily managed [3]. Sensor node battery is neither replaceable nor rechargeable, so the energy conservation has become major challenge for energy-constrained WSN. It has the ability to work without any human intervention. Sensor networks are used in many application areas, like military, agriculture, industry, target tracking, data collection, rescue missions, national security, monitoring disaster prone areas, managing inventories, health care, home security, and environmental studies [4, 5].

Limited energy resources of wireless sensor network should be used wisely to prolong sensor node's lifetime. To achieve high energy efficiency and increase the network lifetime, sensor nodes are grouped together to form clusters. Each cluster consists of sensor nodes within the given range. Every cluster would have a leader, often referred to as cluster head (CH), and the other sensor nodes become cluster members (CMs) of that cluster. A CH may be elected by the sensors in a cluster or preassigned by network designer. Clustering technique has numerous advantages, it can localize the route setup, conserves communication bandwidth, avoids redundant messages exchange, cuts on topology maintenance overhead, implements optimized management strategies to enhance network operations, schedules activities in the cluster, prevents medium access collision by limiting redundancy in coverage, and decreases the number of relayed packets by aggregating data collected by sensors in the network [6]. Sensor nodes (cluster members) within the cluster transmit the sensed data to their cluster head, which in turn forwards the aggregated data to the sink node. This communication happens either directly or via multihop path through other cluster heads. In a clustered network, network traffic is composed of intra- and intercluster traffic. Intracluster communication can be single-hop or multihop, similarly intercluster communication also. Previous research has shown that multihop communication between the source and destination is more energy efficient than direct or single-hop communication [7] due to wireless characteristics. However, the hierarchical (clustering) paradigm causes uneven energy consumption between cluster heads (intercluster communication) and cluster members (intracluster communication). To balance this energy expenditure, previous research proposes cluster head rotation mechanism. This technique balances the energy consumption between CHs and its members but not between the CHs in intercluster multihop communication. Cluster heads close to sink node drain their energy faster due to heavy relay traffic and will die sooner than the other cluster heads. This causes reduction in network coverage and network partitioning. This is called hot-spot problem in WSN. To solve this problem several unequal clustering techniques are proposed in the literature [7–12] to balance the energy consumption between the cluster heads. In unequal clustering technique, clusters close to sink node are smaller in size than those are farther away. Thus, the cluster heads close to base station (Sink) can preserve some energy for intercluster communication.

Organizing wireless sensor networks into clusters enables the efficient utilization of the limited energy resources of deployed sensor nodes. However, the problem of unbalanced energy consumption exists, and it is tightly bound to the role and the location of a particular node in the network [11]. Although it is important to continue pursuing novel algorithms and protocols to squeeze the most out of the existing design space, it is equally important to explore other new design paradigms for future sensor networks. Research in the recent past focused on exploring resource abundant and unconstrained energy sources exist in WSN. Base station (BS) is one such resource rich component in WSN. Properties like resource abundance, unlimited processing capability, huge computational ability, bulky memory, physical location accessibility, and, and so forth made BS as a powerful asset for WSN. This approach taps into capabilities at the edge base station, which has not been fully exploited in prior efforts. This expanded design space has the potential to simplify various algorithms and protocols on a sensor node, thus offering new possibilities to drive down the size and cost of sensor nodes [13]. Several edge-based routing protocols [13–15] are proposed in the literature to shift control overhead burden from sensor node to BS.

In this paper, we consider a new edge-based design space proposed at [16], for sensor networks with the aim of conserving energy resource of a sensor node. We propose a zone-based routing protocol (ZBRP) for WSN based on the considered novel design space. ZBRP considers the features of unequal clustering and edge-based routing capabilities for better network resource utilization. It aims to achieve balanced energy consumption in both intra- and intercluster communication and extends network lifetime by reducing overall communication cost in the network.

## 2. Related Work

Many clustering algorithms and cluster based routing protocols are proposed for WSN. In the recent past, many algorithms are proposed towards effective data communication and data processing with optimal resource usage in the WSN. In this section, we review some of the most effective routing algorithms of WSN.

Low energy adaptive clustering hierarchy (LEACH) [17] is one of the most popular distributed cluster-based routing protocols for WSN. Each node has a certain probability to become cluster head per round, and the task of being a cluster head is rotated among the nodes. LEACH is highly successful in distributing load uniformly across the network. But its single hop routing does not serve the requirement of real world applications. Also, the randomized CH selection method of LEACH fails to maintain uniform load distribution between the cluster heads.

Lindsey and Raghavendra introduced a chain-based clustering routing protocol, PEGASIS [18]. This is considered as an improvement over LEACH routing protocol. The main aim of PEGASIS is to minimize the intracluster communication overhead of LEACH protocol. The key idea of PEGASIS is to form chains with closed by neighboring nodes using greedy approach. Each chain chooses a leader node to forward data to BS. Like LEACH, PEGASIS is single hop routing protocol. So, this is not a good choice for large scale networks.

To address hot spot problem, Li et al. introduced an unequal clustering mechanism, energy efficient unequal clustering (EEUC) [9] to balance energy consumption between the cluster heads. EEUC form small clusters near base station and the size increases as the distance progress. Thus the cluster heads close to base station preserve energy for intercluster communication. The author also proposed an energy aware multihop routing protocol for intercluster communication in EEUC mechanism.

Lee et al. have proposed another unequal clustering algorithm, energy-efficient distributed unequal clustering (EEDUC) [10] to create distributed clusters in WSN. EEDUC is an extension to EEUC [9] mechanism. Here also, clusters closer to the base station have smaller size than those farther away from the base station. It considers relay traffic for selecting forwarding CH to forward data towards BS.

Soro and Heinzelman proposed unequal clustering size (UCS) network organization model for WSN [11]. The main aim of UCS is to enhance the network lifetime by distributing the load uniformly among the CHs, whose positions are predetermined. Having BS at center of the network, the CHs are arranged symmetrically in concentric circles in two levels called Layers. Respective clusters in their respective layers are of same size and shape with CHs at center. But, the cluster size and shape differ from layer to layer. The aggregated data from CHs will be delivered to BS through CH to CH communication.

Bai et al. introduced multihop clustering algorithm, power-efficient zoning clustering algorithm for WSN (PEZCA) [12], to extend network lifetime by minimizing energy consumption. It is developed based on two most popular clustering protocols, low-energy adaptive clustering hierarchy (LEACH) [17] and power-efficient gathering in sensor information systems (PEGASIS) [18]. PEZCA divides its network into fan-shaped regions placing BS at center. Each region is considered as a cluster. CH to CH data communication delivers data to BS.

Mao and Hou have introduced a novel edge-based routing protocol, called BeamStar [13] for WSNs. The aim of BeamStar is to reduce size and cost of the sensor node. This protocol utilizes infrastructure potential provided by an edge based nodes to carry out the network operations. It is assumed that the network is equipped with a directional antenna with power control capabilities. Using this, BS can reach any part of the network to provide control information to sensor nodes by varying its transmission power level and beam width. This shifts the control and network management overhead burden from sensor nodes to BS. The power controlled capability base station scans the complete network with different power transmission levels (sector number (SN)) in different angles (ring number (RN)) to provide location information for the nodes. With this location information, sensor nodes can enroute sensed data to BS using controlled broadcasting mechanism. The data is forwarded by using simple forwarding rules provided by BS.

Chen et al. proposed a routing protocol for edge-based WSNs, called CHIRON [14]. It is developed based on one of the most popular hierarchical routing protocols, PEGASIS [18]. Also, it uses the same technique of BeamStar [13] to provide location information for the nodes in the network. It outperforms BeamStar with respect to delay time and network lifetime. CHIRON operates in four different phases. First phase is Group construction phase, where the sensing field is divided into smaller groups using BeamStar methodology. The nodes with same Ids form groups. Chain formation phase is the second phase. Here, PEGASIS chain formation process is used to construct smaller chains. Leader node election phase is the next phase in CHIRON. Node with maximum residual energy is elected as “Leader node” for the current round. Cluster Head (CH) to Cluster Head communication delivers data to destination node (BS). CH selection process repeats in round robin fashion. The last phase is data collection and transmission phase. In this phase, whenever an event occurs, the sensor nodes sense the data form their surroundings. The sensed data will be collected and aggregated by chain leader. The same is forwarded to BS using multihop, leader-by-leader communication. The CHIRON data transmission process is similar to that of PEGASIS [18] protocol.

To overcome the drawbacks of BeamStar [13], Li and Yang proposed a routing protocol for edge-based WSNs, Cluster-based BeamStar (CBS) [15]. CBS also uses the same concept of BeamStar to provide location information for sensor nodes with refined sensing process. CBS outperforms BeamStar in efficient usage of power, internode communication, and scan time. CBS protocol is explained in three phases. In the first phase, locating phase, sensing field is scanned using BeamStar mechanism by adjusting the transmission power level. The second phase is, cluster building up phase. Here it forms clusters with nodes having same Ids. The node with maximum residual energy is elected as cluster head (CH), just like in CHIRON [14]. Data transmission is the last phase in CBS. It uses LEACH [17] protocol to carry out data transmission process. In this phase, CH aggregates the data from the cluster members and forwards the same to BS via intercluster head transmission. New round starts with an advertisement if CHs energy falls below the given threshold. The cluster member with greater residual energy announces itself as a new cluster head for the current round.

## 3. Zone-Based Routing Protocol

Based on two hard-core mechanisms, clustering and network design space, this paper proposes zone-based routing protocol (ZBRP) for wireless sensor networks. The main aim of ZBRP is to elevate sensor network lifetime by minimizing total energy consumption with limited control overhead on sensor nodes in the network. ZBRP creates even size clusters with minimum control overhead on sensor nodes using their location information. It uses random back-off timers to select cluster heads in each data forwarding round in the network field. Using simple and realistic multihop data forwarding model, ZBRP achieves hot-spot free sensor network with uniform energy consumption among cluster heads.

### 3.1. Network Model

This paper considers a sensor network with *n* sensor nodes deployed uniformly over a circular filed within the radius *R*. The base station is located at center of the network to manage and collect data from the network field. The following assumptions are made before we discuss the proposed work.

1. All the sensor nodes are homogeneous with same capabilities.
2. Nodes are not equipped with GPS (Global Positioning System) capable unit and location unaware.
3. Every node is capable to change its transmission power level depends on the distance to the receiver.
4. Network has continuous data to send.
5. Links are symmetric. Based on RSSI (Received Signal Strength Indication), any node can compute the approximate distance to another node for a given transmission power.

### 3.2. Problems

In a cluster-based wireless sensor networks, cluster heads spend their energy for intracluster communication and intercluster communication [7]. The amount of energy consumed in intracluster communication depends on cluster size. Energy consumption rises with number of sensor nodes in a cluster. The majority of clustering algorithms from literature create even size clusters. Cluster heads of even size clusters tend to consume uniform amount of energy for intracluster communication [9]. Since sensor nodes have limited transmission range, they transmit information to sink node using multihop data transmission model. In data transmission phase, cluster heads act as relay nodes in data forwarding routes to deliver data between source and destination. During this inter cluster communication process, the cluster heads closer to the base station are burdened with heavy relay traffic and will die much faster than far away cluster heads. This raises hot spot problem and reduces network lifetime. To overcome hot-spot problem, unequal clustering technique [9, 10, 12] is proposed in the literature. Unequal clustering mechanism creates uneven size clusters in the network where size of the cluster increases with base station distance. The idea behind creating smaller clusters near BS is to preserve some energy for inter-cluster communication. Unequal clustering mechanism avoids hot spot problem but introduces several other problems into the network. It is successful in achieving uniform energy dissipation among CHs but not between cluster members and cluster heads.

Problems with unequal clustering technique are listed below.

1. Since cluster size rises with base station distance, unequal clustering creates huge clusters as the network size increases and leads to coverage and connectivity issues in the network.
2. Unequal clustering mechanism creates different number of clusters in irregular sizes as there is no control on percentage of CHs it selects in each data transmission round which causes imbalanced energy consumption among sensor nodes in the network.
3. Cluster head selection process involves high control overhead.
4. Dynamic nature in cluster size does not guarantee fully connected network.

To overcome the pitfalls listed above, zone-based routing protocol (ZBRP) is proposed in this paper. ZBRP combines the advantages of equal and unequal clustering techniques to form clusters in the network. Its primary goal is to elevate sensor network's lifetime by minimizing control overhead and total energy consumption in the network.

### 3.3. Network Design Space

**Figure 1:**
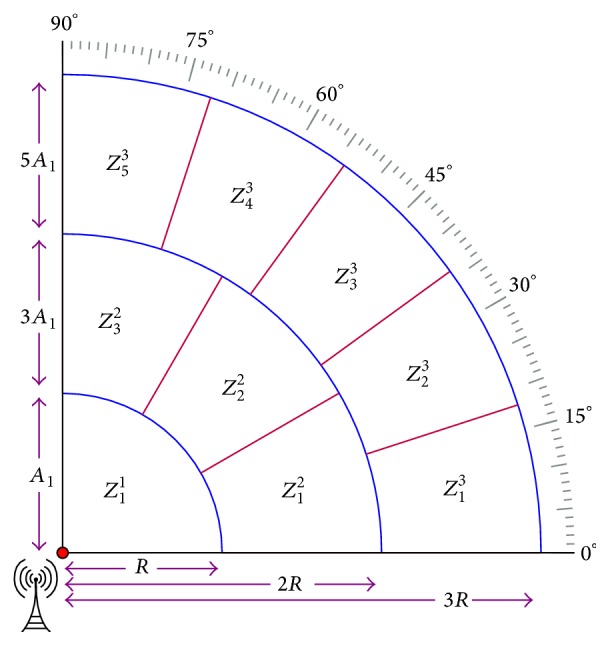
Novel network organization mechanism, where *Z*_*j*_^*i*^ represents *j*th partition of *i*th ring, called Zone. From Figure 1, it is noted that each zone has equal area. These equally spaced zones are used to form even size clusters in the network. This paper considers both traditional (equal) and unequal clustering methods to investigate WSN behavior.

To organize sensor nodes into clusters and to provide identities for sensor nodes, this paper considers network organization scheme proposed in [16]. It is assumed that the base station in the network is equipped with power controlled capability directional antenna. The directional antenna provides location identity to every sensor node in the network by varying its transmission power level in different directions. Using these identities, the sensor nodes are organized into hierarchical clusters except the nodes from first ring. This arrangement is made to avoid relay traffic burden on first ring CH and the nodes from this ring communicate with BS directly.  Figure 1 illustrates the energy-efficient network organization considered in this paper.

### 3.4. Clustering Algorithm

In network initialization phase, base station broadcasts an advertisement to all the sensor nodes in the network. Based on received signal strength each node calculates its distance with BS [12]. To find neighbor nodes, every sensor node advertises its zone id with transmission radius *r*. Number of signals that a node receives form its zone members give neighborhood count. In the first data forwarding round, each node starts a timer (*T*) defined as the follows:

(1) $$\begin{array}{c} \beta =\frac{d\left(S,\text{BS}\right)}{{\text{NH}}_{\text{count}}}\\ T=\frac{1-\alpha }{\beta }, \end{array}$$

where *α* is a random value between (0, 1), *d*(*S*, BS) is distance between source node *S* and base station BS, and number of neighborhood nodes NH_count_. Parameter *β* helps each zone to find a sensor node which has grater neighborhood with smaller distance to base station. Node which clears the timer *T* will announce itself as a CH for that zone and the remaining nodes join the CH by sending a join message. After cluster formation, each cluster member receives TDMA (time division multiple access) slot to send information to its CH, whereas sensor nodes from first ring get TDMA slots form the base station since they communicate with base station directly. Cluster heads aggregate data received from its cluster members and forwards it as a single fixed length data packet to downstream CH towards base station.

From second round onwards, each node starts back-off timer as given in (4) to compete for cluster head position:

(2) $$\begin{array}{c} p=\frac{E_{\text{init}}-E_{\text{re}}}{R_{\text{num}}-1}, \end{array}$$

(3) q=EseEre∗1−pNHcount,

(4) $$\begin{array}{c} T=\frac{1}{1-q}, \end{array}$$

where *E*_re_ is residual energy, *E*_init_ is initial energy, *R*_num_ is round number, *E*_se_ is node spent energy, and NH_count_ is number of neighbor nodes.

Each sensor node starts its timer using its local information to compete for cluster head position. For cluster head competition, sensor nodes use communication cost (*p*) as a primary parameter. The neighborhood count (NH_count_) of sensor node plays an important role in selecting a cluster heads in the network. This parameter allows ZBRP to choose a sensor node with maximum visibility in each zone to guarantee good coverage and makes cluster head selection process distributive. Sensor node with lower communication cost having greater coverage scope will clear the timer first. This method helps the network to rotate cluster heads uniformly among sensor nodes in a zone-based network. Node which clears the timer *T* announces itself as CH and other sensor nodes stop timers and join the CH. Unlike other clustering algorithms [9, 10, 12], the proposed work uses only local information to select cluster heads in each data forwarding round to minimize control overhead in the network. In the initial stage, distance with base station is considered as a primary parameter and in later stages communication cost plays prominent role in identifying distributed cluster heads in the network.

**Figure 2:**
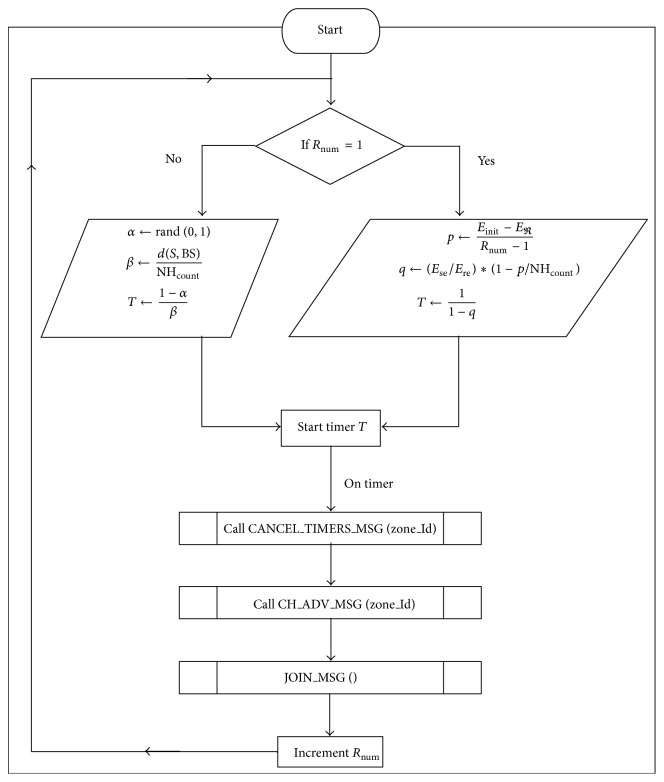
ZBRP flow chart.

The Pseudocode 1 explains the pseudo code of proposed clustering mechanism and Figure 2 represents the flow chart for the same.

### 3.5. Multihop Communication

Every CH aggregates the data received locally and then sends the information to BS via multihop data forwarding model. CH to CH inter cluster communication mechanism delivers data to BS. The relaying CH is primarily selected based on its location information [16]. Every cluster head keep track number of messages it relayed in previous rounds and is used during relay node selection process. Prior to each data forwarding round, cluster heads announces their location id, residual energy, distance with base station, and number of messages it relayed. During data transmission process, source cluster head utilizes these details to find its relay node to avoid selecting few cluster heads often to forward data and promotes invariable energy consumption among cluster heads in the network. Node from downstream closer to base station having greater residual energy with lesser amount of messages forwarded will be selected as a relay node for current data transmission process. Source cluster head always broadcasts its forwarding message with its relaying cluster head id. This indicates other cluster heads to update the message counter of the relaying node for future relay node selection process. When data reaches second ring, the CHs communicate this information directly to BS, whereas sensor nodes from first ring communicate sensed data directly to base station. For first ring sensor nodes BS allots TDMA slots in network deployment phase to receive information directly from them. With proper relay cluster head selection process, ZBRP achieves uniform energy consumption among data forwarding cluster heads and avoids hot spot problem in the network.

## 4. Simulation Results

In this section, the proposed work is evaluated via simulation using Castalia Simulator [19]. The behavior of proposed work is studied in terms of energy consumption and network lifetime. The proposed work ZBRP is compared with a well-known cluster-based routing protocol LEACH [17] and with an unequal cluster-based routing protocol PEZCA [12]. Simplified radio hardware energy dissipation model shown in [17] is used in this paper. The simulation parameters used for experimental work are given in Table 1.

**Figure 3:**
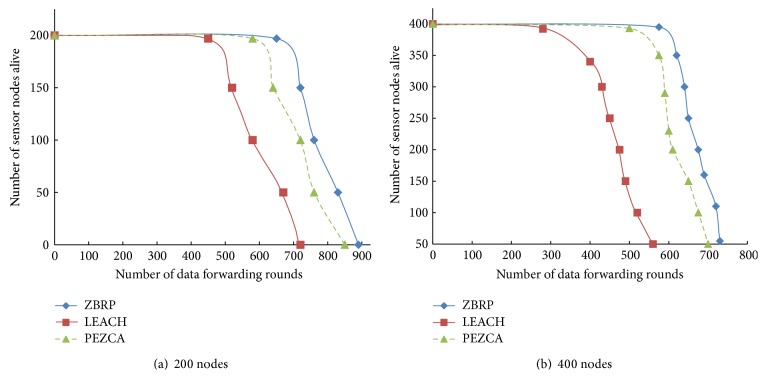
Number of sensor nodes alive with number of rounds.

Figure 3 depicts the number of sensor nodes alive in the network versus number of rounds for LEACH, PEZCA, and ZBRP.  Figure 3(a) represents the number of live nodes in a 200 node network and Figure 3(b) represents the number of live nodes in a 400 node network with number of data forwarding rounds. From Figure 3, it is noted that the proposed routing protocol, ZBRP, minimizes energy consumption with its distributed cluster head selection process and helps the sensor nodes live longer than LEACH and PEZCA. Also, it is observed from the figure that the number of sensor nodes die out is substantial in LEACH and PEZCA, whereas it is gradual in ZBRP.

**Figure 4:**
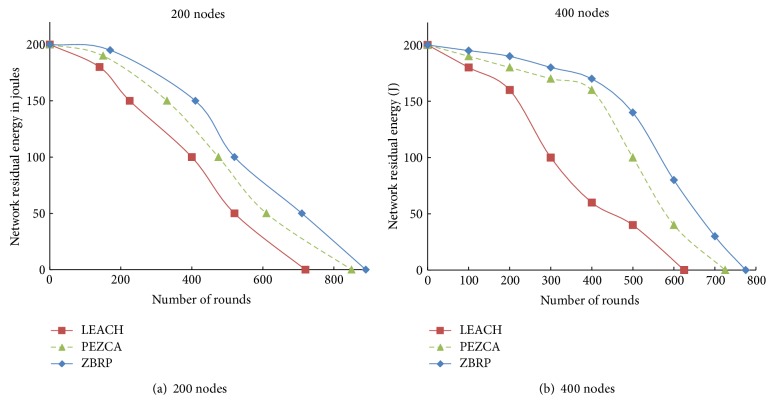
System residual energy with number of rounds.

Figure 4 shows network residual energy of three routing protocols, LEACH, PEZCA, and ZBRP. From the figure it is evident that ZBRP performs better than LEACH and PEZCA in terms of energy consumption. ZBRP achieves invariable energy dissipation with its multihop communication mechanism among cluster heads across the network and helps the network to conserve its resources.

**Figure 5:**
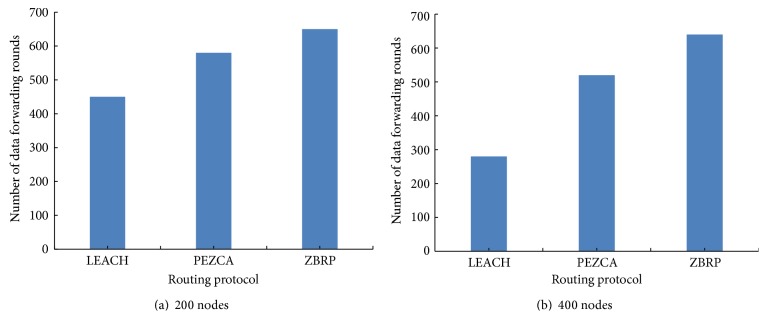
Network lifetime with number of rounds.

Figures 5(a) and 5(b) show network lifetime of the three algorithms when 1% nodes dead in 200 nodes and 400 nodes network, respectively. It is realized from Figure 5 that ZBRP improves network lifetime compared to LEACH and PEZCA. With its distributed cluster head selection mechanism, ZBRP selects cluster heads uniformly across the zone-based network and saves energy of every individual sensor node. The ZBRP multihop communication mechanism enables invariable energy dissipation among cluster heads and promotes hot spot free network with enhanced sensor network lifetime.

## 5. Conclusion

In this paper, we introduce a novel energy-efficient cluster-based routing protocol, called zone-based routing protocol, for edge-based WSNs. To avoid hot spot problem in traditional clustering methods, unequal clustering mechanism has been introduced. Though it is a widely accepted solution for hot spot problem, it has scalability issues with varied network sizes. Since proper network design space could solve the inconsistent load distribution, we have investigated different network organization models to address hot spot problem. Therefore, proper network design space with efficient clustering technique could solve the issue. We have proposed a zone-based routing protocol for the network model described in [16]. The design space considered plays prominent role in forming equal clusters with proper sensor node distribution across the network. From the simulation results, it is inferred that, the proposed routing protocol distributes the load uniformly with proper cluster formation across the network and enhances the sensor network lifetime.

## Figures and Tables

**Pseudocode 1:**
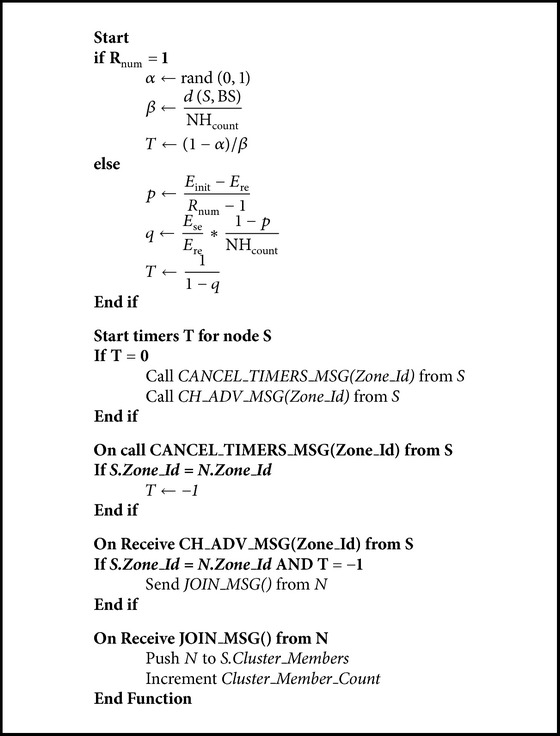
ZBRP pseudocode.

**Table 1:** Simulation parameters.

Parameter	Value
Simulation area	100 m × 100 m & 200 m × 200 m
Simulator	Castalia [19]
Base station	(0, 0)
Number of sensor nodes	400 & 200
Node deployment type	Uniform
Initial energy	2000 joules
Energy consumed to transmit or receive (*E*_elec_)	50 nJ/bit
Transmit amplifier (*E*_amp_)	100 pJ/bit/m^2^
Data packet size	2000 bits
Packet rate	1 per second
Number of runs	10
Simulation time	1500 seconds
Round time	50 seconds
Radius (*R*)	100 m & 200 m
